# Sericin-mediated improvement of dysmorphic cardiac mitochondria from hypercholesterolaemia is associated with maintaining mitochondrial dynamics, energy production, and mitochondrial structure

**DOI:** 10.1080/13880209.2022.2055088

**Published:** 2022-03-29

**Authors:** Kitiya Rujimongkon, Sumate Ampawong, Duangnate Isarangkul, Onrapak Reamtong, Pornanong Aramwit

**Affiliations:** a Department of Pharmacy Practice, Faculty of Pharmaceutical Sciences and Center of Excellence in Bioactive Resources for Innovative Clinical Applications, Chulalongkorn University, Bangkok, Thailand; b Proteomics Research Team, National Omics Center, National Science and Technology Development Agency, Pathumthani, Thailand; c Department of Tropical Pathology, Faculty of Tropical Medicine, Mahidol University, Bangkok, Thailand; d Department of Microbiology, Faculty of Science, Mahidol University, Bangkok, Thailand; e Department of Molecular Tropical Medicine and Genetic, Faculty of Tropical Medicine, Mahidol University, Bangkok, Thailand, and;; f The Academy of Science, The Royal Society of Thailand, Bangkok, Thailand

**Keywords:** Silkworm, cocoon, proteomics, mitochondrial proteome, heart

## Abstract

**Context:**

Sericin is a component protein in the silkworm cocoon [*Bombyx mori* Linnaeus (Bombycidae)] that improves dysmorphic cardiac mitochondria under hypercholesterolemic conditions. This is the first study to explore cardiac mitochondrial proteins associated with sericin treatment.

**Objective:**

To investigate the mechanism of action of sericin in cardiac mitochondria under hypercholesterolaemia.

**Materials and methods:**

Hypercholesterolaemia was induced in Wistar rats by feeding them 6% cholesterol-containing chow for 6 weeks. The hypercholesterolemic rats were separated into 2 groups (*n* = 6 for each): the sericin-treated (1,000 mg/kg daily) and nontreated groups. The treatment conditions were maintained for 4 weeks prior to cardiac mitochondria isolation. The mitochondrial structure was evaluated by immunolabeling electron microscopy, and differential mitochondrial protein expression was determined and quantitated by two-dimensional gel electrophoresis coupled with mass spectrometry.

**Results:**

A 32.22 ± 2.9% increase in the percent striated area of cardiac muscle was observed in sericin-treated hypercholesterolemic rats compared to the nontreatment group (4.18 ± 1.11%). Alterations in mitochondrial proteins, including upregulation of optic atrophy 1 (OPA1) and reduction of NADH-ubiquinone oxidoreductase 75 kDa subunit (NDUFS1) expression, are correlated with a reduction in mitochondrial apoptosis under sericin treatment. Differential proteomic observation also revealed that sericin may improve mitochondrial energy production by upregulating acetyl-CoA acetyltransferase (ACAT1) and NADH dehydrogenase 1α subcomplex subunit 10 (NDUFA10) expression.

**Discussion and conclusions:**

Sericin treatment could improve the dysmorphic mitochondrial structure, metabolism, and energy production of cardiac mitochondria under hypercholesterolaemia. These results suggest that sericin may be an alternative treatment molecule that is related to cardiac mitochondrial abnormalities.

## Introduction

Mitochondria are an important source of energy production in cells, especially in the heart, which is a high metabolic demand organ. The high metabolic demand of myocardial cells is essential to support the blood circulatory system. Abnormalities of mitochondria in cardiomyocytes are a cause of heart failure (Chen and Knowlton 2010; Zhou and Tian 2018). Hypercholesterolaemia is a major risk factor related to cardiovascular disease (Burkhardt 2015). High cholesterol levels and low abundance of high-density lipoprotein (HDL) in serum are related to mitochondrial dysfunction through increasing reactive oxygen species (ROS) and causing myocardium dysfunction (McCommis et al. 2011; Bhatti et al. 2017).

High serum cholesterol is one of the factors that leads to myocardial and cardiac mitochondrial degeneration (Ampawong et al. 2017a). Ultrastructural observations of mitochondria have revealed four mitochondrial stages from a normal to a severely degenerated structure. The normal stage involves a structure containing double membranes (inner and outer membranes) covering the intermembrane space. The inner membrane forms pore-like structures termed crista junctions in the mitochondrial matrix. The swelling stage involves the first dysmorphic structure, which includes an increased size, distensions of the intercellular matrix, and partial disappearance of cristae. The spheroid stage is characterised by the complete loss of cristae and the formation of multiple cysts in the matrix. In the final stage, the ghost stage, the membrane disappears, leaving granular and electrodense material (Mariappan et al. 2007; Ampawong et al. 2017a, 2017b). Dysmorphic cardiac mitochondria under hypercholesterolemic coupled with hyperglycaemic conditions have revealed different numbers of mitochondria between the dysmorphic and normal stages (Ampawong et al. 2017a). This evidence suggests the possibility that high serum cholesterol levels are related to the structure of cardiac mitochondria, are associated with mitochondrial dysfunction and result in organ failure, especially in the heart.

Mitochondrial dynamics is a physiological condition that involves maintaining mitochondrial activity and energy production and depends on the metabolism of the cell. The crucial properties of dynamics, including fusion and fission, are associated with apoptosis (Suen et al. 2008). Optic atrophy 1 (OPA1) and dynamin-related protein 1 (DRP1) are machinery proteins that are involved in the fusion and fission of mitochondria, respectively. Downregulation of DRP1 delays apoptotic cell death (Frank et al. 2001). Conversely, reduced OPA1 expression induces the release of cytochrome c and apoptosis (Olichon et al. 2003). Impairment of mitochondrial dynamic proteins has been implicated in several heart diseases, such as myocardial infarction (Ong et al. 2010), cardiac hypertrophy (Pennanen et al. 2014), and heart failure (Ashrafian et al. 2010). Therefore, differential expression of mitochondrial dynamic proteins can be used to describe proteins involved in mitochondrial function and apoptosis.

Sericin is a natural molecule found in the silkworm *Bombyx mori* Linnaeus (Bombycidae) cocoon and possesses various biochemical properties, including antityrosinase, anti-lipid peroxidation, and antioxidant activities (Kato et al. 1998; Dash et al. 2008; Aramwit et al. 2010a, 2010b; Takechi et al. 2014). Several methods, including urea, citric acid, sodium carbonate, and high temperature coupled with high-pressure extraction, have been used to characterise the amino acid content, structure, and biochemical properties of sericin (Aramwit et al. 2010a). Sericin has been used in several biomedical applications, such as for wound healing and as a biomaterial essential for tissue engineering (Aramwit and Sangcakul 2007; Aramwit et al. 2013; Lamboni et al. 2015). Sericin extracted using high-temperature and high-pressure methods have been used as a food supplement and have been shown to be able to significantly reduce serum cholesterol in a hypercholesterolemic rat model (Ampawong et al. 2017a, 2017b).

The improvement of mitochondrial dysmorphia in the liver and heart of hypercholesteremic rats induced by sericin is due to its ability to improve the antioxidative stress response and maintain mitochondrial dynamics. In a recent experiment involving liver mitochondrial proteomics analysis of hypercholesterolemic rats treated with sericin, we detected altered expression of several proteins involved in mitochondrial homeostasis (Ampawong et al. 2018). Four classes of proteins related to mitochondrial homeostasis in hypercholesterolaemia were found to be altered. These classes included apoptosis, autophagy, energy production and antioxidative stress proteins. In our previous study, sericin was also shown to improve the mitochondrial architecture in the hearts of hypercholesterolemic rats; however, the mechanism by which this improvement occurred was not determined.

This study aimed to investigate the effect of sericin on cardiac mitochondria in a hypercholesterolemic rat model by observing the structure of cardiac muscle contraction, cardiac mitochondrial dynamics, and cardiac mitochondrial proteomics to elucidate the treatment effect of sericin. The proteomic results provided information on the pathways related to the effect of sericin. Our findings may provide information regarding the specific mechanism by which sericin treatment improves mitochondrial function in cardiac tissue.

## Materials and methods

### Sericin extraction

Sericin was prepared from *B. mori* cocoons provided by Chul Thai Silk Co., Ltd. (Phetchabun Province, Thailand). Sericin was extracted under high temperature and high pressure as described by Aramwit et al. (2010a, 2010b). Briefly, cocoon shells were autoclaved in distilled water at 120 °C for 1 h. The supernatant containing sericin was collected, filtered, and lyophilised. Sericin powder was solubilised in distilled water before use in experiments.

### Animal experimental protocol

#### Animal ethics statement

The animal study protocol was approved by the Animal Care and Use Committee, Faculty of Medicine, Chulalongkorn University, (Approval No. 16/2558). All procedures were performed in accordance with the Animals for Scientific Purposes Act, B.E. 2558 (A.D. 2015), Thailand. All animals were housed under strict hygienic conditions with controlled temperature and humidity and a 12 h light/dark cycle. The animals were fed *ad libitum* with a standard diet (Perfect Companion Ltd., Thailand).

#### Hypercholesterolaemia induction and treatment

Female Wistar rats (eight weeks old and weighing 200–300 g) were obtained from the National Laboratory Animal Centre, Mahidol University (NLAC-MU). The rats were fed 6% cholesterol-containing chow (Perfect Companion Ltd., Thailand) ad libitum for 6 weeks before the experiments and represented a diet-induced hypercholesterolaemia model (Ampawong et al. 2017a). Hypercholesterolemic rats were separated into two groups, each containing six rats. The control and sericin-treated groups were given distilled water and 1,000 mg/kg sericin daily, respectively, by oral gavage for four weeks. This dose of sericin has already been confirmed to improve the structure of heart mitochondria under hypercholesterolemic conditions (Ampawong et al. 2017b). All rats were humanely sacrificed using an overdose of isoflurane by inhalation.

#### Specimen collection

To measure the blood marker profiles of each experimental rat, blood specimens were collected from each rat by cardiac puncture and centrifuged at 1,500 *g* for 15 min. Serum (6 samples/group) was separated, and the markers of serum lipids including cholesterol and HDL were measured by the Quality Control Division of NLAC-MU.

Heart specimens were collected and immediately separated and stored in two different solutions. For mitochondrial extraction, heart specimens were kept in homogenising buffer (0.32 M sucrose, 1 mM EDTA, 10 mM Tris-HCl, pH 7.4). For histopathology and electron microscopy analyses, the specimens were immersed in 10% neutral buffer formalin (NBF) and in 2.5% glutaraldehyde in 0.1 M sucrose phosphate buffer pH 7.4 (SPB), respectively. To avoid technical artefacts in the cell and organelle architecture studies, all processes were performed on ice.

#### Mitochondrial extraction

The mitochondrial extraction protocol has been described previously by Ampawong and colleagues (2018). Heart specimens from each treatment group (6 hearts/group) were pooled by similar weights and cut into small pieces. The specimens were homogenised in ice-cold homogenising buffer using a glass Potter Elvehjem tissue grinder. A motor-driven Teflon pestle was moved up and down at 900 rpm until the sample was homogenised. The homogenates were centrifuged at 1,000 × *g* for 5 min at 4 °C, and the supernatant was removed. The mitochondrial pellets were collected and washed by centrifugation at 1,000 × *g* for 5 min at 4 °C in homogenising buffer several times; then, the washed pellets were resuspended in ice-cold equilibration buffer [250 mM sucrose, 5 mM KH_2_PO_4_, 10 mM Tris-HCl, 2 mg/mL bovine serum albumin (BSA), pH 7.2].

### Proteomic studies

#### Two-dimensional gel electrophoresis (2DE)

Mitochondrial proteins were extracted using lysis buffer (7 M urea, 2 M thiourea, 4% CHAPS, 1% v/v protease inhibitor) and were measured by a protein assay (Bio–Rad®, USA) and a spectrophotometer (NanoDrop-1000, Thermo Scientific, USA). The protocol of Isarangkul and colleagues was used (Isarangkul et al. 2015). Briefly, the first dimension separated the proteins by isoelectric focussing. Each mitochondrial protein sample was loaded onto an Immobiline™ Drystrip-immobilized pH gradient strip (13 cm, pH 3–10 NL, GE Healthcare^®^, USA) using the passive rehydration method for 12 h. Isoelectric focussing as the first dimension was performed using an Ettan IPGphor instrument (GE Healthcare^®^, USA) at 20 °C. Reduction and alkylation of the proteins on the gel strip were performed in equilibration buffer (75 mM Tris-HCl, pH 8.8; 6 M urea; 30% glycerol; 2% sodium dodecyl sulphate (SDS); 0.002% bromophenol blue) containing 1% dithiothreitol (DTT) for 15 min; the strip was then incubated in equilibration buffer containing 2.5% iodoacetamide for 15 min. The second dimension separated the proteins by molecular mass. The equilibrated gel strips were loaded onto a 12.5% SDS gel, sealed with agarose sealing solution and subjected to a current using a vertical slab gel electrophoresis unit (SE 600 Chroma Hoefer^®^) at 15 mA/gel for 30 min and then at 30 mA/gel until bromophenol blue reached the bottom of the gel. The gel was fixed in fixing solution (40% ethanol, 10% acetic acid) for 2 h, stained with Flamingo™ Fluorescent gel stain (Bio-Rad^®^, USA) for 18 h and washed with distilled water. The 2DE experiment was performed in triplicate.

#### Image analysis

Fluorescently stained gels were scanned using a Typhoon Trio Variable Mode Imager (GE Healthcare^®^, USA) under a 532 nm green laser and 555 nm long-pass emission filter. Image Master 2 D Software (GE Healthcare^®^, USA) was used to detect, quantify, and compare the spots based on their optical density values. The differentially expressed spots with significant (*p* < 0.05) differences according to an analysis of variance (ANOVA) were chosen for protein identification.

#### In-gel tryptic digestion

The selected spots were collected from the 2DE gels. The gels were destained with 50% acetonitrile until colourless and then dehydrated in 100% acetonitrile (HPLC grade, Merck^®^, USA). Proteins were reduced in a DTT solution (10 mM DTT in 100 mM ammonium bicarbonate (NH_4_HCO_3_) for 1 h at 56 °C and alkylated in iodoacetamide (55 mM iodoacetamide in 100 mM NH_4_HCO_3_) for 45 min at room temperature with protection from light. The gels were washed using 100 mM NH_4_HCO_3_ for 45 min at 4 °C to remove all traces of the abovementioned solutions. A trypsin solution and 12.5 ng/µL trypsin (Promega, USA) were added to the gels and incubated at 37 °C overnight. Tryptic-digested peptides were extracted using 20 mM NH_4_HCO_3_ buffer and 5% formic acid in 50% acetonitrile for 20 min at room temperature. The digested peptides were processed using a SpeedVac concentrator (Thermo Scientific, USA). The dried peptides were subjected to mass spectrometry analysis.

#### Protein identification

To identify proteins, the digested peptides were dissolved in 0.1% formic acid and then subjected to the UltiMate® 3000 Nano LC System (Dionex, USA) with an Acclaim PepMap RSLC C18 75 μm × 15 cm column (Thermo Scientific, USA) in stationary phase. Mobile phase solutions A and B consisted of 0.1% formic acid in 2% acetonitrile and 0.1% formic acid in 80% acetonitrile, respectively. The initial mobile phase was maintained at 4% solution B for 5 min, linearly increased to 45% solution B over 25 min, held for 5 min and, finally, returned to the initial condition over 10 min. A maxis UHR-TOF (Bruker, USA) was used to record peptide spectra over the mass range of m/z 50–2200 in the positive electrospray ionisation mode. DataAnalysis Software, Version 3.4, (Bruker, USA) was used to convert the mass spectra into a Mascot generic file (.mgf). Mascot, Version 2.4.1, (Matrix Science, UK) was used to identify mitochondrial proteins from the .mgf file. Proteins were obtained from the SwissProt Database (SwissProt 20161111). The search parameter setting for methionine oxidation was set as a fixed modification, and that for carbamidomethylation of cysteine was set as a variable modification. The identified proteins with significant scores (*p* < 0.05) were reported.

### Morphological studies

#### Histopathology and immunofluorescence of heart tissue sections

Fixed heart specimens (6 hearts/group) were used for standard tissue processing and were cut to a thickness of 5 µm. The sections were deparaffinized in xylene, hydrated in a series of graded ethanol solutions and heat-retrieved to enhance their antigenicity in citrate buffer, pH 6.0. To determine histopathological changes in cardiac muscle between hypercholesterolemic rats with or without sericin treatment, image analysis was used to semiquantify striated cardiac muscle from toluidine blue-stained tissue. Colour images (10 images/heart) were acquired using light microscopy (BX41, Olympus^®^, Japan) and a digital camera (DP20, Olympus^®^, Japan) at 400X magnification. The percentage of striated area per muscular area field was quantified using ImageJ (NIH, USA).

The expression of acetyl-CoA acetyltransferase (ACAT1) and NADH dehydrogenase 1 alpha subcomplex subunit 10 (NDUFA10) in heart tissue sections was determined by immunofluorescence. Deparaffinized sections (as previously described) were subjected to immunofluorescence detection using the VectaFluor Duet Immunofluorescence Double Labelling Kit with the DyLight 488 anti-rabbit antibody (Vector, USA). Primary antibodies, polyclonal rabbit anti-ACAT1 and polyclonal rabbit anti-NDUFA10 (MyBioSource, USA) antibodies, were incubated with separate tissue sections. Then, the DyLight 488 anti-rabbit secondary antibody was applied to the sections. Nuclei were counterstained using Vectashield Antifade mounting medium with DAPI (Vector, USA). Immunolocalization of these two markers was determined under a fluorescence microscope (BX41, Olympus^®^, Japan).

#### Transmission electron microscopy of mitochondria

Transmission electron microscopy was used to examine the ultrastructure of mitochondria. Purified mitochondria from the pooled heart extract as previously described were fixed in 200 μL of 2.5% glutaraldehyde in 0.1 M SPB. Secondary fixation was performed using 1% osmium tetroxide, followed by dehydration with graded ethanol, infiltration in a series of LR white resin (EMS^®^, USA), embedding in pure LR white (EMS^®^, USA) and polymerisation at 60 °C for 48 h. The mitochondrial pellets were cut to a thickness of 100 nm. The sections were stained with lead citrate and uranyl acetate. The mitochondrial structure was examined under a transmission electron microscope (TEM; model HT7700, Hitachi, Japan). Dysmorphic mitochondria were counted and compared between sericin-treated and untreated rats.

#### Immunogold labelling of mitochondria

Immunogold labelling was performed to observe the mitochondrial structure and quantify the expression of the proteins of interest. Six protein markers (MyBioSource, USA) were used as primary antibodies, including the fusion marker rabbit polyclonal antioptic atrophy 1 (OPA1), the fission marker rabbit polyclonal antidynamin-related protein 1 (DRP1), the energy marker rabbit polyclonal antihaloacid dehalogenase-like hydrolase domain-containing protein 3 (HDHD3), the apoptotic marker mouse polyclonal anti-NADH-ubiquinone oxidoreductase 75 kDa subunit (NDUFS1), the fatty acid oxidation marker rabbit polyclonal anti-ACAT1, and the antioxidative stress marker rabbit polyclonal anti-NDUFA10.

The mitochondrial sections, processed as previously described, were incubated with a 1:50 dilution of primary antibodies for 1 h before applying gOAT anti-rabbit or anti-mouse IgG conjugated with ultrasmall (3–10 nm) gold particles (EMS^®^, USA). The sections were washed using 0.1% BSA in PBS several times between each step. To enhance the visibility of gold particle labelling, a silver enhancement kit (Aurion R-Gent SE-EM kit, EMS^®^, USA) was used after washing the sections with distilled water. Finally, the sections were stained with lead citrate and uranyl acetate before TEM examination. The number of labelled gold particles was counted as immunolabeling per mitochondria (lpm) at each stage of cardiac mitochondria: normal, swelling, spheroid, and ghost. Each experimental group was evaluated using at least 100 mitochondria. Approximately 20 mitochondria were used as representatives for each stage, including the normal, swelling, spheroid, and ghost stages.

### Statistics

GraphPad Prism^®^ version 5 was used for data processing and statistical analysis. Differences in quantitative data were compared using Student’s *t*-test and are reported as the mean ± SEM. Statistical significance was indicated by a *p* value less than 0.05 (*p* < 0.05).

## Results

### Sericin improved cardiac muscle contraction under hypercholesterolaemia

The present study assessed hypercholesterolemic rats divided into two experimental groups: sericin-treated and untreated. The serum marker revealed a cholesterol level of 66.34 ± 5.32 mg/dL in healthy rats. For hypercholesterolemic rats, cholesterol levels were measured at 77.66 ± 4.41 and 234.16 ± 3.34 mg/dL in the sericin-treated and untreated groups, respectively. Rats fed a high-cholesterol diet had 3-fold higher cholesterol levels than rats fed a normal diet. Sericin-fed rats showed decreased serum cholesterol levels compared to healthy rats. In contrast, sericin increased HDL (72.0 ± 1.57 mg/dL) compared with that in nontreated (50.0 ± 1.35 mg/dL) hypercholesterolemic rats but did not reach the level found in healthy rats (84.11 ± 7.22 mg/dL). These results suggested that sericin was mainly effective in reducing serum cholesterol.

**Figure 1.**
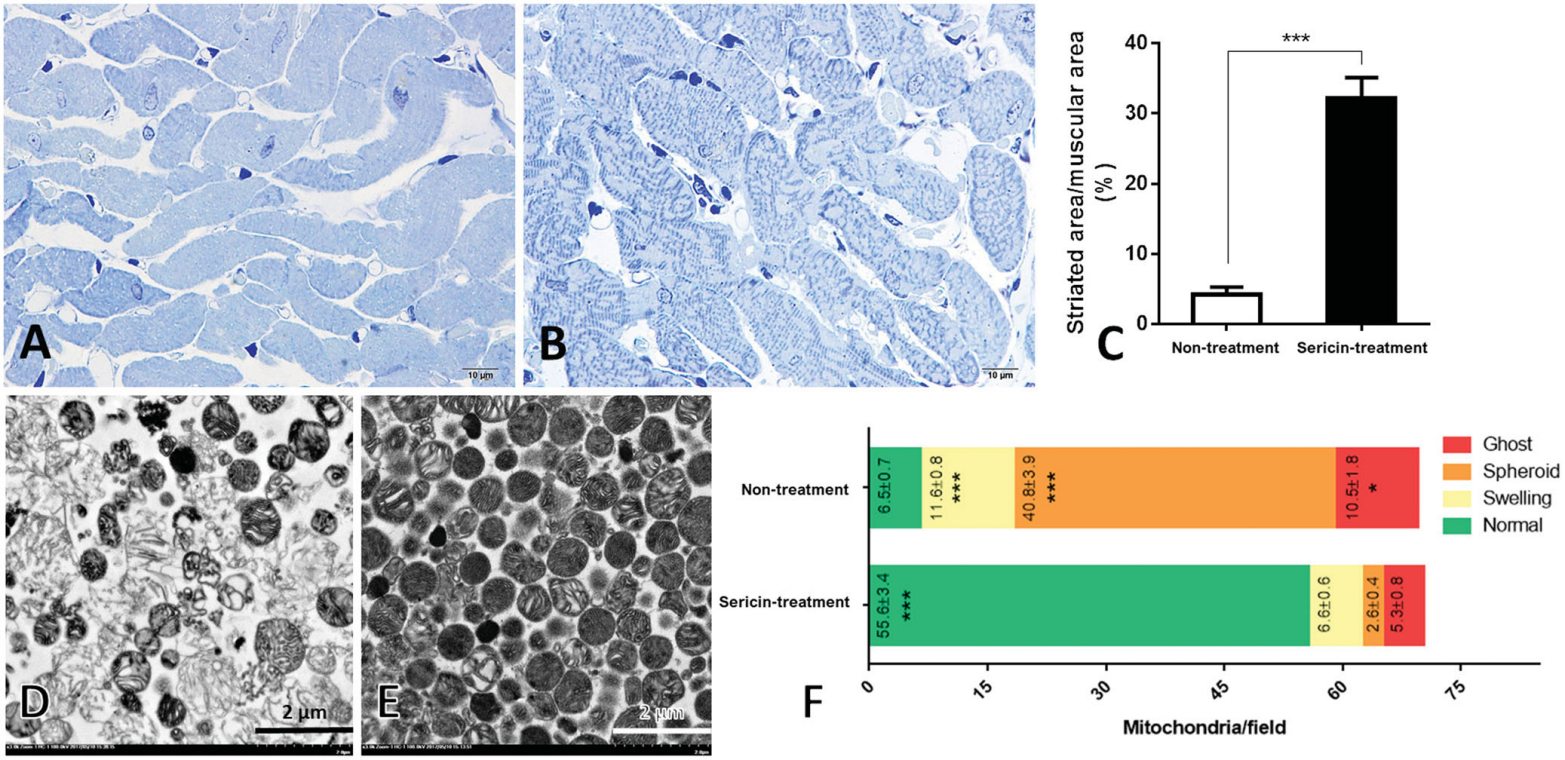
Histopathology of cardiac muscle and ultrastructure of heart mitochondria: Histopathology of cardiac muscle from hypercholesterolemic rat model was observed through heart non-treated (A) and sericin-treated (B) rats. Percent of striated area per muscular area was demonstrated in the bar graph (C). Electron microscopy was illustrated mitochondrial morphology from heart non-treated (D) and sericin-treated (E) rats. Bar graph (F) represented gold labelling by mean ± SEM comparing each mitochondrial stage; normal (green), swelling (yellow), spheroid (orange), and ghost (red), between non-treated and sericin-treated rats.

Histopathology of heart tissue from hypercholesterolemic rats with and without sericin treatment was performed using cardiac muscle comparison (Figure 1A,B). The percent striated area per muscular area field, as an indication of cardiac muscle contraction activity, was larger in the sericin-treated group (32.22 ± 2.9%) than in the untreated group (4.18 ± 1.11%) (Figure 1C). This result indicates that sericin has the potential to improve cardiac muscle morphology under hypercholesterolemic conditions, suggesting that sericin produced beneficial effects on cardiac architecture.

### Effect of sericin on the recovery of cardiac mitochondrial structure

The ultrastructure of cardiac mitochondria was demonstrated in four stages in both sericin-treated and untreated hypercholesterolemic rats (Figure 1D,E). The average counts of mitochondria per field (mpf) in the normal stage significantly differed between the sericin-treated (55.66 ± 3.4 mpf) and untreated (6.5 ± 0.7 mpf) groups (Figure 1F), with a 9-fold increase in the normal architecture of mitochondria after sericin treatment. Conversely, significantly more dysmorphic structures (swelling, spheroid, and ghost) were found in the untreated group than in the sericin-treated group. The spheroid stage was the most common form of mitochondria in the untreated group (40.8 ± 3.9 mpf) compared with the sericin-treated group (2.6 ± 0.4 mpf). This result revealed the effects of sericin on the recovery of the cardiac mitochondrial structure, as it led to a significantly increased number of mitochondrial structures at the normal stage and reduced numbers at all three dysmorphic stages.

### Sericin alteration of mitochondrial dynamic protein expression

**Figure 2.**
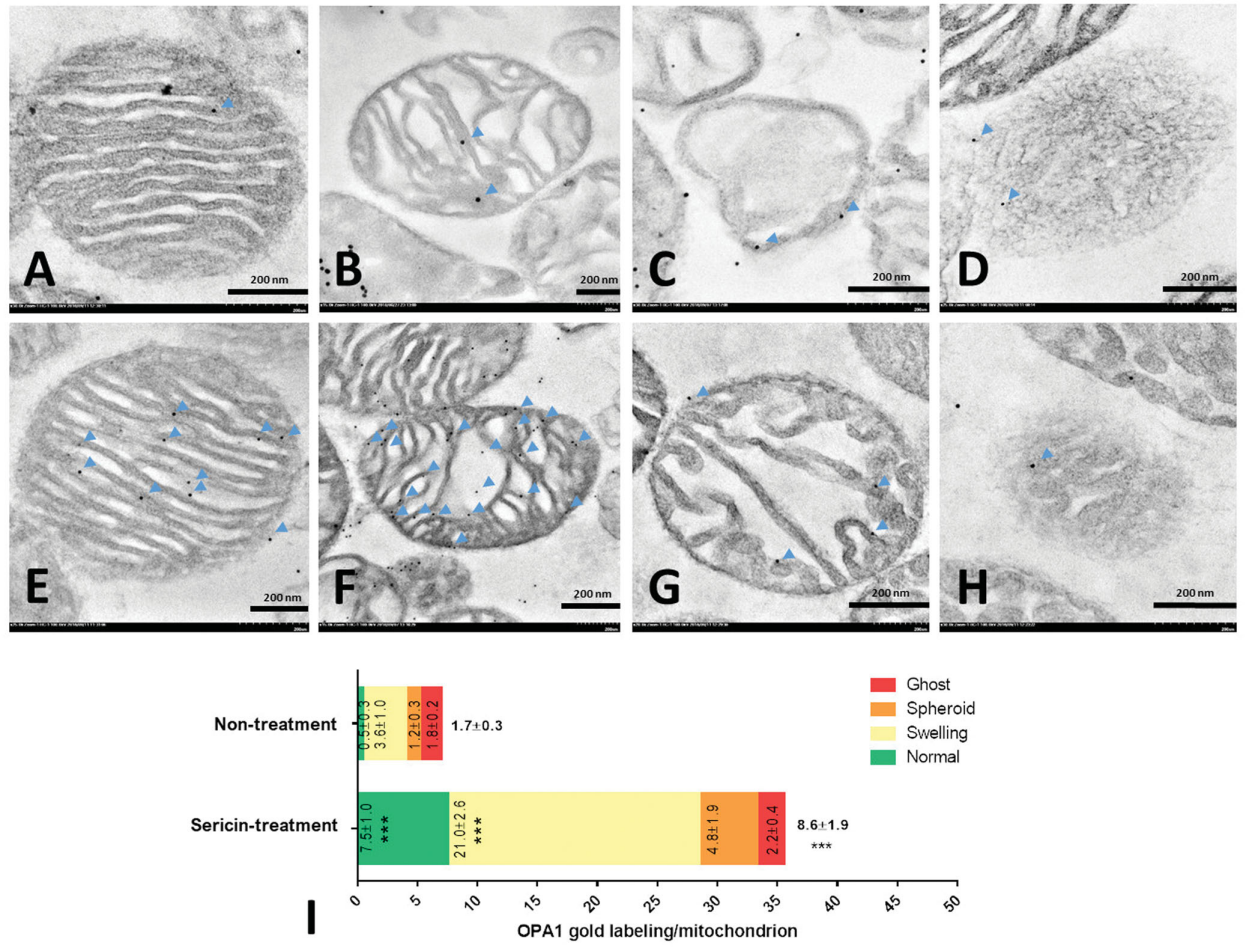
OPA1 immunogold labelling in extracted heart mitochondria was observed by electron microscopy: Non-treatment (A–D) and sericin-treatment (E–H) with OPA1 gold labelling (arrow) at heart mitochondrial stages: normal (A,E), swelling (B,F), spheroid (C,G), and ghost (D,H). Bar graph (I) is demonstrated of gold labelling by mean ± SEM comparing each mitochondrial stage; normal (green), swelling (yellow), spheroid (orange), and ghost (red), between non-treated and sericin-treated rats.

**Figure 3.**
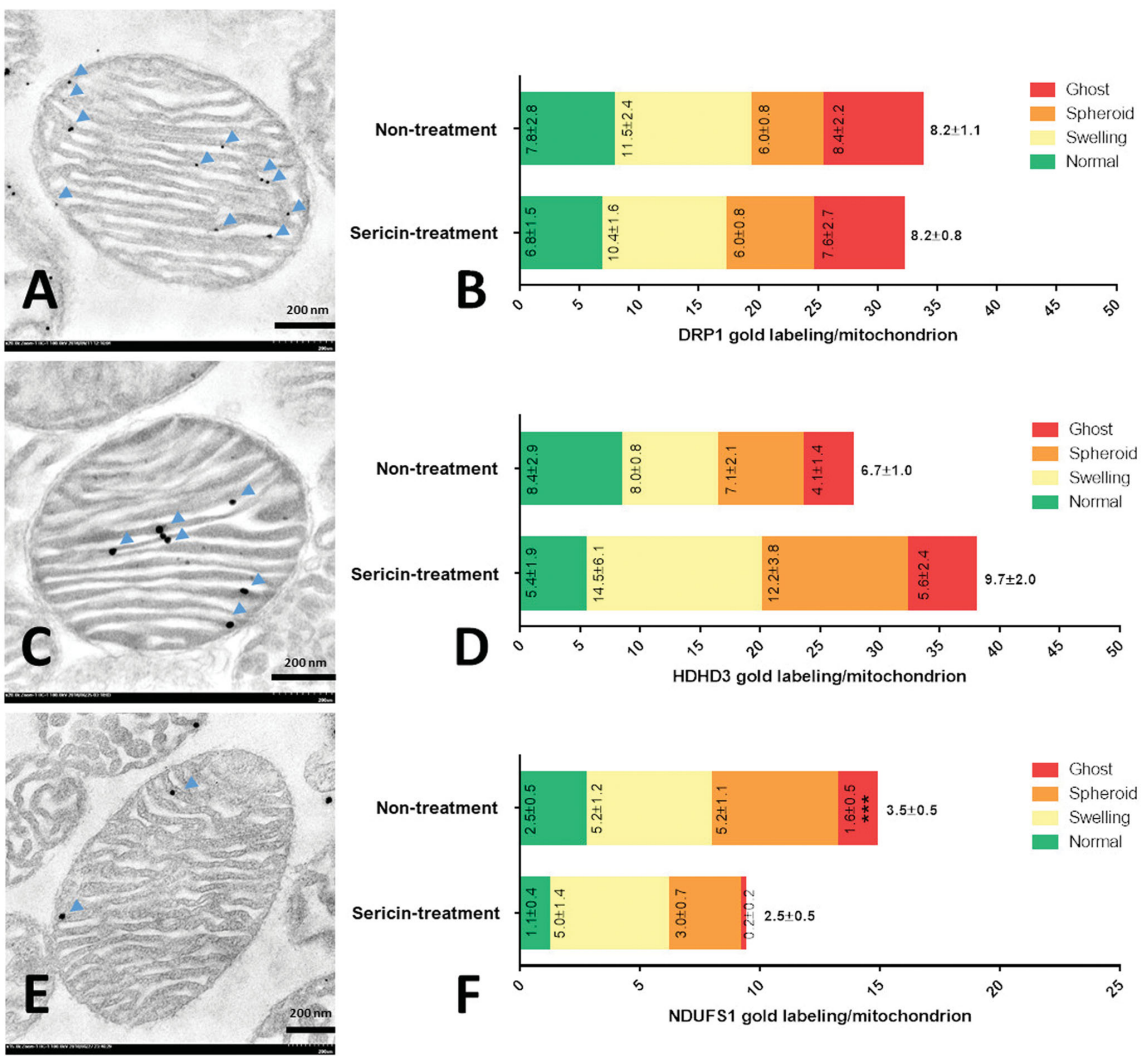
DRP1, HDHD3, and NDUFS1 immunogold labelling in extracted heart mitochondria were observed by electron microscopy: Normal stage mitochondria with gold labelling (arrow) were represented in DRP1 (A), HDHD3 (C), and NDUFS1 (E). The bar graph is demonstrated gold labelling of DRP1 (B), HDHD3 (D), and NDUFS1 (F) by mean ± SEM comparing each mitochondrial stage: normal (green), swelling (yellow), spheroid (orange), and ghost (red), between non-treated and sericin-treated rats.

The mitochondrial dynamic proteins OPA1 and DRP1 were observed in various cardiac mitochondrial stages in each treatment in this experiment. The average expression of OPA1, a fusion marker, by immunogold labelling revealed a significant 5-fold increase in OPA1 in sericin-treated mitochondria (8.6 ± 1.9 lpm in the sericin-treated group and 1.7 ± 0.3 lpm in the untreated group; Figure 2). Regarding specific structural expression, the normal and swelling stages exhibited significantly higher expression of OPA1, with 15-fold and 6-fold increases with sericin treatment (7.5 ± 1.0 lpm in the normal stage and 21.6 ± 2.6 lpm in the swelling stage) compared with untreated mitochondria (0.5 ± 0.3 lpm in the normal stage and 3.6 ± 1.0 lpm in the swelling stage), respectively. Similar amounts of the fission marker DRP1 were detected in both the sericin-treated and untreated groups (Figure 3A,B). These results indicate that mitochondrial fusion was increased by the activity of sericin in the heart, but sericin had no effect on mitochondrial fission.

### Effects of sericin on cardiac mitochondrial energy maintenance and apoptosis

The effects of sericin on the expression of an energy maintenance marker (HDHD3) and an apoptosis marker (NDUFS1) were investigated. Immunogold labelling of HDHD3 demonstrated that its expression was slightly increased in the sericin-treated group (9.7 ± 2.0 lpm) compared to the untreated group (6.7 ± 1.0 lpm), but without significance (Figure 3C,D). Regarding the different patterns of NDUFS1 labelling, the average detection was found to decrease from 3.5 ± 0.5 lpm in the untreated group to 2.5 ± 0.5 lpm in the sericin-treated group, but without significance (Figure 3E,F). However, NDUFS1 expression in the ghost form, the last dysmorphic mitochondrial stage, was significantly decreased by sericin treatment (0.2 ± 0.2 lpm) compared with the untreated group (1.6 ± 0.5 lpm). These results suggested that sericin did not significantly improve energy maintenance via HDHD3 activity. However, sericin inhibited the progression of apoptosis at the last stage of dysmorphic mitochondria according to the reduction in reduced NDUFS1.

### Alterations in cardiac mitochondrial energy production, metabolism, and structure in the sericin-treated hypercholesterolemic rat model

**Figure 4.**
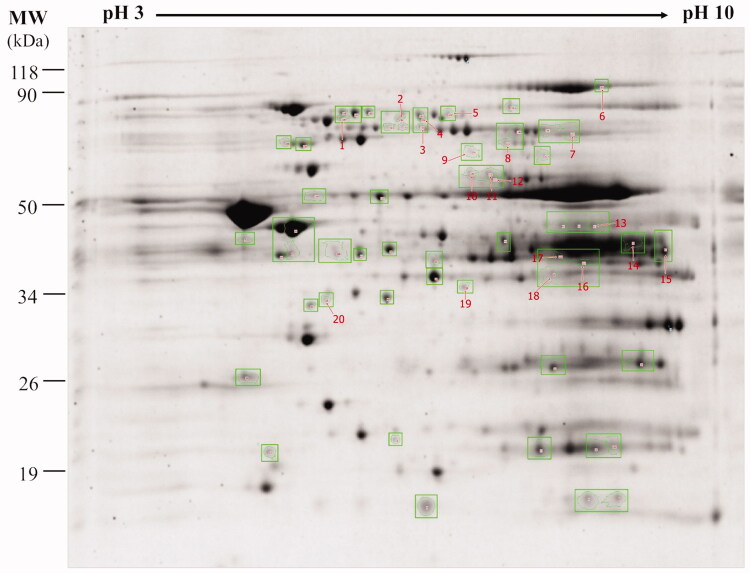
Two-dimensional gel electrophoresis (2DE) of heart mitochondria from sericin-treated hypercholesterolemic rats: Mitochondrial proteins were separated by isoelectric point of proteins (pI) and molecular weight (MW) in 12.5% SDS-PAGE. The gel was stained by flamingo fluorescent and visualised by Typhoon Trio Variable Mode Imager.

In total, using 2DE analysis, 255 protein spots were identified from the untreated and sericin-treated cardiac mitochondrial protein expression profiles. Fifty-nine spots were found to differ between sericin-treated and untreated hypercholesterolemic rats. Twenty spots showed significantly different protein expressions (Figure 4). These proteins were categorised into three groups: (i) eight spots were only present in sericin-treated rats (Table 1), (ii) six spots were upregulated in sericin-treated rats (Table 2), and (iii) six spots were downregulated in untreated rats (Table 3). The identities of seventeen proteins from twenty spots were determined by mass spectrometry. Fourteen differential proteins with activity in mitochondrial energy production encompassed six functions: electron transport chain [(succinate dehydrogenase ubiquinone flavoprotein subunit (SDHA), cytochrome b-c1 complex subunit 2 (UQCRC2), NADH dehydrogenase [ubiquinone] 1 α subcomplex subunit 10 (NDUFA10), and electron transfer flavoprotein-ubiquinone oxidoreductase (ETFDH)]; fatty acid oxidation [hydroxysteroid dehydrogenase-like protein 2 (HSDL2), acetyl-CoA acetyltransferase (ACAT1), and very-long-chain specific acyl-CoA dehydrogenase (ACADYL)]; pyruvate metabolism [acetyl-coenzyme A synthetase 2-like (ACSS1), dihydrolipoyl dehydrogenase (DLD), and isocitrate dehydrogenase [NADP] (IDH2)]; Krebs cycle [fumarate hydratase (FH) and aconitate hydratase (ACO2)]; amino acid metabolism [aspartate aminotransferase (GOT2)] and iron-sulfur cluster biogenesis [stress-70 protein (HSPA9)]. Another three mitochondrial proteins were identified with roles in metabolism [propionyl-CoA carboxylase β chain) (PCCB)], mitochondrial structure [actin, aortic smooth muscle (ACTA2)], and unknown activity [β-2-glycoprotein 1 (APOH)].

**Table 1.** Proteins present in heart mitochondria post sericin-treated hypercholesterolemic rats.

Spot No.	Intensity		ANOVA	Gene name	Protein name	Score	MW	pI	%Sequence coverage	Mitochondria related function
	Treatment	Non-treatment								
1	0.0398	ND	0.0046	HSPA9	Stress-70 protein	43	73970	5.87	7	Iron-sulfur cluster biogenesis (Alfadhel et al. 2017; Lill and Freibert 2020), Cardioprotection (antiapoptosis) (Gao et al. 2015)
2	0.1927	ND	0.0045	Hsdl2	Hydroxysteroid dehydrogenase-like protein 2	286	58649	5.85	10	Fatty acid oxidation (Gronemeyer et al. 2013)
3	0.0833	ND	0.0358	Hsdl2	Hydroxysteroid dehydrogenase-like protein 2	532	58649	5.85	17	
4	0.1104	ND	0.0359	Sdha	Succinate dehydrogenase [ubiquinone] flavoprotein subunit	133	72596	6.75	3	Electron transport chain (Renkema et al. 2015), Mutation is cause of cardiomyopathy (Levitas et al. 2010)
5	0.1433	ND	0.0234	Acss1	Acetyl-coenzyme A synthetase 2-like	101	75317	6.51	6	Pyruvate metabolism, Oxidation under ketogenic condition (Fujino et al. 2001)
6	0.0230	ND	0.0098	Dld	Dihydrolipoyl dehydrogenase	81	54574	7.96	9	Pyruvate metabolism (Igamberdiev et al. 2004), Cardioprotection (antioxidative stress) (Igamberdiev et al. 2004)
7	0.0334	ND	0.0064	Fh	Fumarate hydratase	528	54714	9.06	15	Krebs cycle (Ajalla Aleixo et al. 2019)
8	0.1222	ND	0.0023	Idh2	Isocitrate dehydrogenase [NADP]	2797	51330	8.88	30	Pyruvate metabolism (Smolkova and Jezek 2012), Cardioprotection (antioxidative stress) (Benderdour et al. 2004)

MW: molecular weight, pI: isoelectric point.

**Table 2.** Upregulated heart mitochondrial protein post-sericin-treated hypercholesterolemic rats.

Spot No.	Intensity		ANOVA	Gene name	Protein name	Score	MW	pI	%Sequence coverage	Mitochondria related function
	Treatment	Non-treatment								
1	0.2177	0.0298	0.0371	Uqcrc2	Cytochrome b-c1 complex subunit 2	2574	48423	9.16	33	Electron transport chain (Fernandez-Vizarra and Zeviani 2018)
2	0.0344	0.0089	0.0462	Uqcrc2	Cytochrome b-c1 complex subunit 2	1492	48423	9.16	31	
3	0.1460	0.0270	0.0112	Acat1	Acetyl-CoA acetyltransferase	396	45009	8.92	13	Fatty acid oxidation (Fukao et al. 1998), Prevention of atherosclerosis (Wakabayashi et al. 2018)
4	0.9752	0.3709	0.0414	Got2	Aspartate aminotransferase	597	47683	9.13	19	Amino acid metabolism (Hoffmann and Solter 2008)
5	0.8757	0.3776	0.0063	Ndufa10	NADH dehydrogenase [ubiquinone] 1 alpha subcomplex subunit 10	355	40753	7.64	18	Electron transport chain (Pandey et al. 2017), Cardioprotection (antioxidative stress) (Pandey et al. 2017)
6	0.2003	0.0931	0.0091	Acta2	Actin, aortic smooth muscle	743	42381	5.23	19	Mitochondrial structure (Boldogh and Pon 2006), Cardioprotection (antiapoptosis) (Boldogh and Pon 2006)

mtDNA: mitochondrial DNA, MW: molecular weight, pI: isoelectric point.

**Table 3.** Downregulated heart mitochondrial protein post-sericin-treated hypercholesterolemic rats.

Spot No.	Intensity		ANOVA	Gene name	Protein name	Score	MW	pI	%Sequence coverage	Mitochondria related function
	Treatment	Non-treatment								
1	0.0393	0.1112	0.0022	Aco2	Aconitate hydratase	820	86121	7.87	15	Krebs cycle (Fernandes et al. 2015)
2	0.0301	0.1171	0.0062	Acadvl	Very long-chain specific acyl-CoA dehydrogenase	1869	71047	9.01	28	Fatty acid oxidation (Le et al. 2000), Cardioprotection (bioenergetics and Ca2+ homeostasis) (Cecatto et al. 2018)
3	0.0386	0.2527	0.0055	Etfdh	Electron transfer flavoprotein-ubiquinone oxidoreductase	363	69010	7.33	18	Electron transport chain (Zhang et al. 2006)
4	0.0330	0.2117	0.0066	Apoh	Beta-2-glycoprotein 1	37	34316	8.59	5	Unknown function, Deficiency is cause of myocardial infarction in clinical evidence (Ranzolin et al. 2004)
5	0.1650	0.2352	0.0014	Pccb	Propionyl-CoA carboxylase beta chain	84	59216	7.19	8	Metabolism (Jiang et al. 2005)
6	0.0356	0.2001	0.0061	Pccb	Propionyl-CoA carboxylase beta chain	106	59216	7.19	6	

MW: molecular weight, pI: isoelectric point.

Considering that protein expression is related to cardiac mitochondrial function, nine pathways were determined to be affected by sericin treatment. Four of the proteins expressed only in the sericin-treated group had functions specific to pyruvate metabolism (3/4) and iron-sulfur cluster biogenesis (1/4). Two pathways, amino acid metabolism and mitochondrial structure were found to be upregulated. The altered proteins specific to metabolism and unknown functions only included downregulated proteins. The other three functions, the Krebs cycle, fatty acid oxidation, and electron transport chain, showed mixed expression, with increased and decreased protein expression in sericin-treated hypercholesterolemic rats.

Cardioprotective proteins were also found, with two main functions reported in antioxidative stress (consistent with the upregulation of three proteins, DLD, IDH2, and NDUFA10, and the downregulation of ACO2) and antiapoptosis (consistent with the increase in HSPA9 and the decrease in ACTA2 after sericin treatment).

### Upregulation of protein-regulated energy production in cardiac mitochondria of the sericin-treated rat model

**Figure 5.**
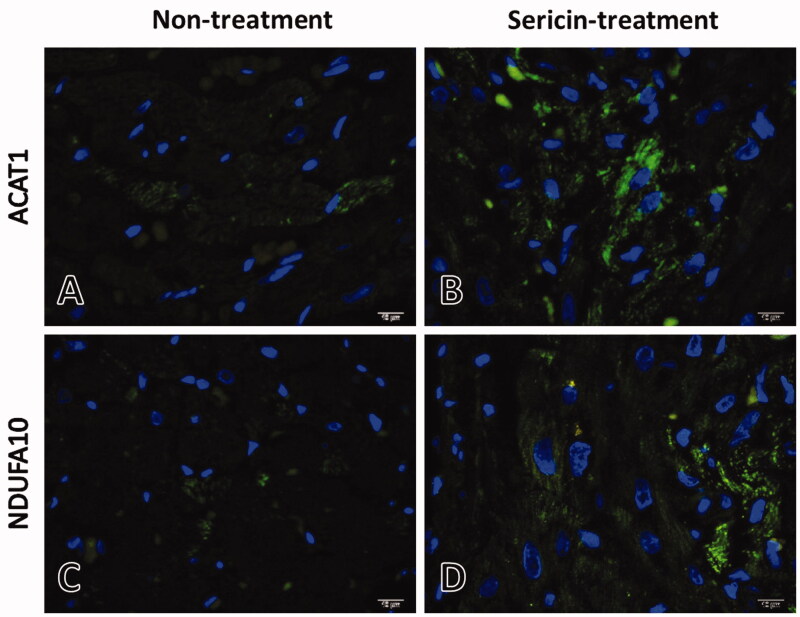
ACAT1 and NDUFA10 in heart tissue of non-treated (A,C) and sericin-treated (B,D) rats under hypercholesterolaemia: ACAT1 and NDUFA10 were stained with DyLight 488 (green), DNA (blue), and visualise by fluorescent microscopy.

**Figure 6.**
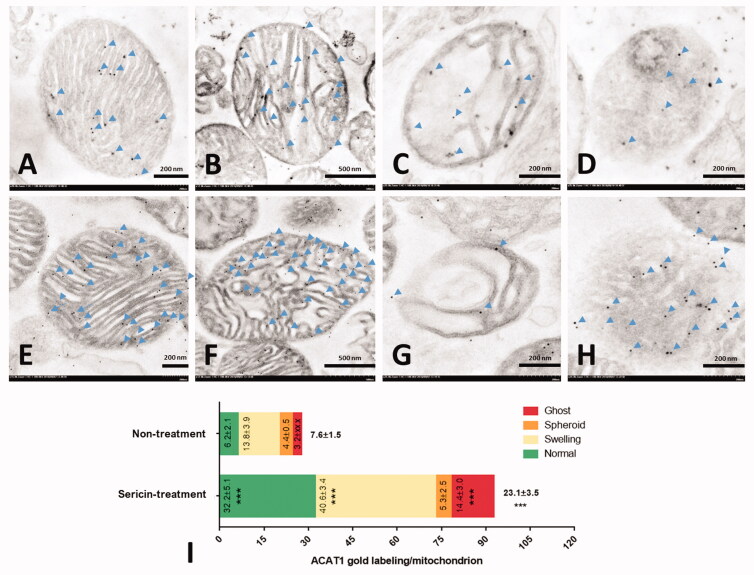
ACAT1 immunogold labelling in extracted heart mitochondria was observed by electron microscopy: Non-treatment (A–D) and sericin-treatment (E–H) with ACAT1 gold labelling (arrow) at heart mitochondrial stages: normal (A,E), swelling (B,F), spheroid (C,G), and ghost (D,H). Bar graph (I) is demonstrated of gold labelling by mean ± SEM comparing each mitochondrial stage; normal (green), swelling (yellow), spheroid (orange), and ghost (red), between non-treated and sericin-treated rats.

**Figure 7.**
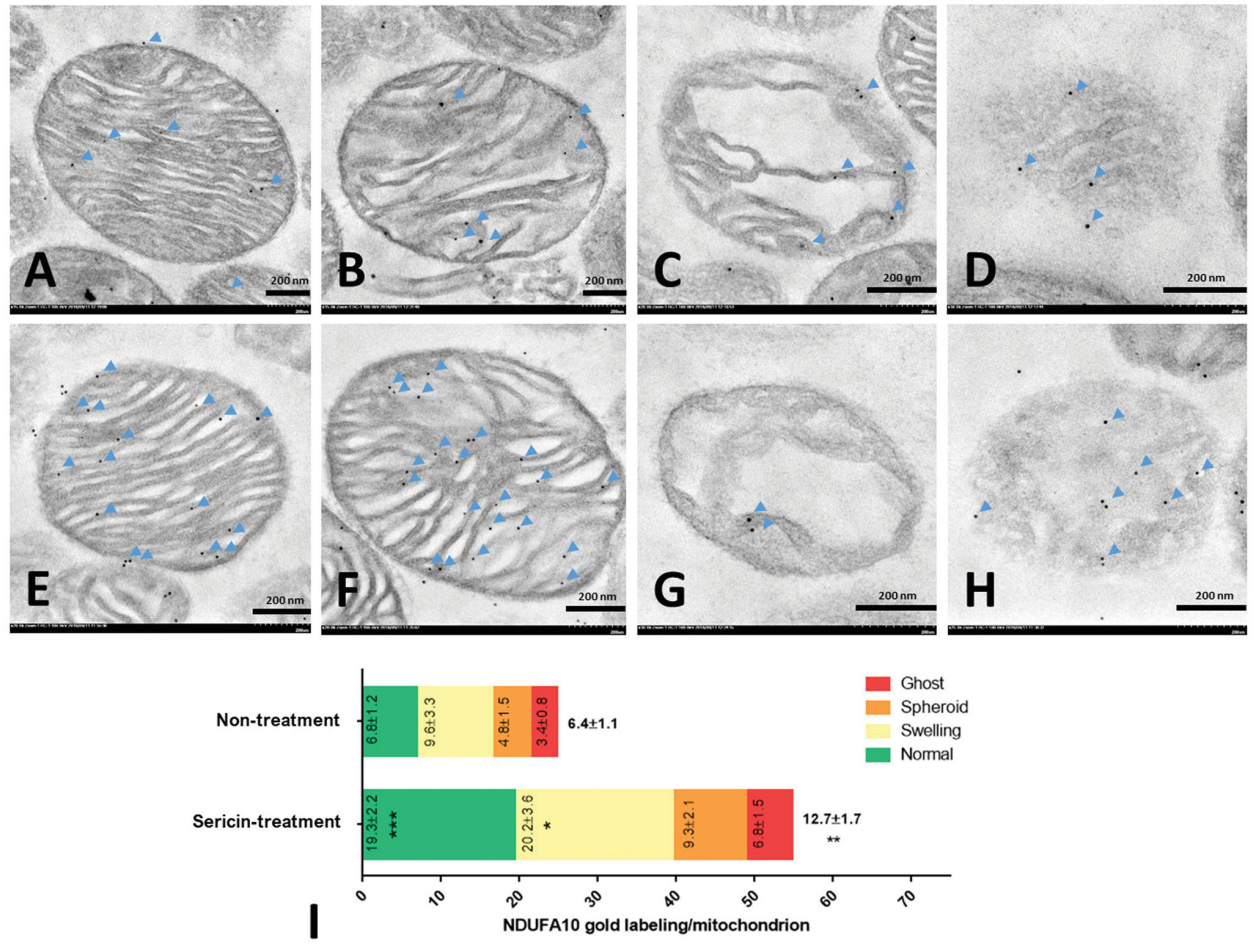
NDUFA10 immunogold labelling in extracted heart mitochondria was observed by electron microscopy: non-treatment (A–D) and sericin-treatment (E–H) with NDUFA10 gold labelling (arrow) at heart mitochondrial stages: normal (A,E), swelling (B,F), spheroid (C,G), and ghost (D,H). Bar graph (I) is demonstrated of gold labelling by mean ± SEM comparing each mitochondrial stage; normal (green), swelling (yellow), spheroid (orange), and ghost (red), between non-treated and sericin-treated rats.

Immunofluorescence and immunogold labelling of two upregulated proteins from the protein expression profile of the sericin-treated group, ACAT1 and NDUFA10, were performed to validate the results of the proteomics experiment. The immunofluorescence microscopy results showed the signals of these two proteins separately in sericin-treated heart tissue sections. Weak signals for the ACAT1 and NDUFA10 proteins were observed under the untreated condition, while strong signals were observed for two proteins under the sericin treatment condition (Figure 5). Using the high-resolution technique, immunogold labelling coupled with transmission electron microscopy was performed to characterise the cardiac mitochondrial structure and quantify the expression of these two proteins in each mitochondrial stage. The results showed that the average expression of ACAT1 exhibited a 3-fold increase in the sericin-treated group (23.1 ± 3.5 lpm) compared with the untreated group (7.6 ± 1.5 lpm; Figure 6). In normal structures, ACAT1 labelling presented a 5-fold increase in sericin-treated mitochondria (32.2 ± 5.1 lpm) compared with untreated mitochondria (6.2 ± 2.1 lpm). Mitochondria with degenerative structures also showed a significant 3-fold increase in ACAT1 labelling in the swelling and ghost forms in the sericin-treated group compared to the untreated group. Another upregulated protein from the proteomic study, NDUFA10, also showed a significant 2-fold increase in NDUFA10 gold labelling in the sericin-treated group (12.7 ± 1.7 lpm) compared with the untreated group (6.4 ± 1.1 lpm) (Figure 7). The amounts of NDUFA10 labelling in mitochondria in the normal and swelling forms significantly increased by 3-fold and 2-fold in sericin-treated animals (19.3 ± 2.2 lpm in the normal stage and 20.2 ± 3.6 lpm in the swelling stage) compared with untreated animals (6.8 ± 1.2 lpm in the normal stage and 9.6 ± 3.3 lpm in the swelling stage), respectively. These two validation techniques, immunofluorescence microscopy of heart tissue and immunogold labelling of mitochondrial extracts, confirmed the protein expression results of the proteomics study.

## Discussion

Cardiovascular disease affects the heart and blood vessels and leads to abnormal functions of both. Based on a proteomics approach, differential proteomes have been found to include many altered proteins related to heart disease. This approach is a potentially useful method with various applications for cardiovascular disease, including biomarker identification (Gallego-Delgado et al. 2005), target therapeutics (Lam et al. 2016), and disease mechanisms (Rüdebusch et al. 2017). One major risk factor of cardiovascular disease is hypercholesterolaemia (Andreadou et al. 2017). Cardiac dysfunction studies based on cardiac gene expression analyses in cholesterol-enriched diet-fed animal models have revealed that hypercholesterolaemia affects energy metabolism, stress proteins, ion channel proteins, structural proteins, and oxidative stress (Puskas et al. 2004; Varga et al. 2013). A differential proteomics analysis study revealed correlations between some mitochondrial proteins (involved in oxidative stress and lipid metabolism) and cardiovascular dysfunction in hypercholesterolaemia (Park et al. 2004). In addition, studying proteomic profiles and dysmorphic mitochondria is useful for observing pathways related to the mitochondrial structure in the hypercholesterolemic rat model (Ampawong et al. 2017a, 2017b). Therefore, investigating the mechanisms related to the mitochondrial structure using ultrastructure coupled with proteomic techniques could be useful for identifying pathways specific to structural changes.

Natural products are used to study therapeutic functions in various cardiovascular diseases (Ooi et al. 2018; Ramanathan et al. 2018). Sericin, a natural protein extract, has therapeutic effects on cell proliferation, oncogenesis, and oxidative stress in animal models (Sasaki et al. 2000). Moreover, dysmorphic mitochondria in the heart under hypercholesterolaemia have been recovered with sericin treatment (Ampawong et al. 2017a). Therefore, sericin may have therapeutic effects on heart dysfunction by improving mitochondrial activity. However, the effect of sericin treatment on mitochondrial cardiac dysfunction remains uncharacterised. Our report is the first to characterise the changes in cardiac mitochondrial proteins using proteomics to determine the mechanisms by which sericin treatment induces positive effects in a hypercholesterolemic animal model.

Histological examination of heart tissue demonstrated that the striated area of cardiac muscle increased in sericin-treated hypercholesterolemic rats. This result suggests that sericin is able to rescue a dysmorphic heart, which may lead to impaired muscular function after hypercholesterolaemia. Hypercholesterolaemia is the cause of heart contractile dysfunction by inducing myocardial oxidative/nitrative stress (Huang et al. 2004; Csont et al. 2007; Varga et al. 2013). Related studies have also shown that the antioxidant marker Nrf-2 is increased in cardiac vascular walls and mitochondria in hypercholesterolemic rats after sericin treatment (Ampawong et al. 2017a). Oxidative stress is one factor that leads to mitochondrial dysfunction (Ott et al. 2007). Moreover, in the present study, a higher number of normal cardiac mitochondria was observed after sericin treatment. Thus, energy production in sericin-treated mitochondria may be more active than that in untreated rats. Therefore, sericin may lead to the recovery of heart contractile function by reducing oxidative stress and maintaining energy production in the heart.

Mitochondrial dynamics control mitochondrial function. Two key proteins representing the fission and fusion of mitochondria are DRP1 and OPA1, respectively. The mitochondrial fusion structure maintains the inner membrane structure and functions to protect cells from apoptosis, while mitochondrial fission is associated with apoptotic cell death (Parone and Martinou 2006). Related studies in a heart failure rat model have shown the relationship between dynamic protein expression and apoptosis. Reduced OPA1 but not DRP1 induces mitochondrial fragmentation, which is associated with apoptosis (Chen et al. 2009). In our study, the results demonstrated that cardiac mitochondria of hypercholesterolemic rats showed no alterations in DRP1 protein expression, whereas increased OPA1 expression was observed after sericin treatment. A caspase substrate in apoptosis, NDUFS1, was significantly reduced at the ghost stage of dysmorphic mitochondria. This evidence indicated that sericin maintained cardiac mitochondrial dynamics by increasing mitochondrial fusion and, consequently, prevented apoptosis. This dynamic action differed from that observed in liver mitochondria in a hypercholesterolaemia rat model; liver mitochondria show decreased DRP1 expression and increased OPA1 expression after sericin treatment (Ampawong et al. 2018), which implies that sericin treatment affects mitochondrial dynamics in different organs in distinct ways.

The expression of HDHD3 and NDUFS1 has been reported to produce an adaptive effect related to the improved hepatic mitochondrial structure under hypercholesterolemic conditions through sericin treatment (Ampawong et al. 2018). HDHD3 plays a role in mitochondrial metabolism (Giguère 2008). Our study showed that the expression of HDHD3 tended to increase but did not significantly lead to a change in cardiac mitochondria after sericin treatment. Conversely, a previous study on liver mitochondrial structures revealed that sericin induces HDHD3 expression in all stages of mitochondria to maintain energy levels (Ampawong et al. 2018). Sericin may have no effect on cardiac mitochondrial metabolism, in contrast to its effects on liver mitochondria. Another protein, NDUFS1, is involved in electron transport chain complex I. NDUFS1 has also been reported to be a caspase substrate in apoptosis (Ricci et al. 2004). In this study, cardiac mitochondria showed no changes in the average expression of NDUFS1 after sericin treatment. Only the ghost stage (the last stage of dysmorphic mitochondria) had significantly reduced NDUFS1 expression after sericin treatment. This result suggests that sericin reduces NDUFS1 expression in ghost-stage mitochondria to prevent apoptosis and is similar to NDUFS1 expression in liver mitochondria, which shows decreased expression after sericin treatment (Ampawong et al. 2018). This evidence implies that sericin improves mitochondrial function after hypercholesterolaemia by an independent function of the affected organ.

A cardiac mitochondrial proteomic study was performed to investigate proteins related to the effects of sericin under hypercholesterolemic conditions. The differential protein profile revealed three classes of proteins related to mitochondrial function: energy production (pyruvate metabolism, fatty acid oxidation, Krebs cycle, and electron transport chain), metabolism (iron-sulfur cluster biogenesis, amino acid metabolism, and mitochondrial metabolism), and mitochondrial structure. Most of the altered proteins identified in this study were involved in energy production. Interestingly, pyruvate metabolism (ACSS1, DLD, and IDH2) and iron-sulfur cluster biogenesis (HSPA9) were exclusively affected by sericin treatment. Cardioprotection has been reported to be an antioxidative stress mechanism of DLD, IDH2, and NDUFA10 (Benderdour et al. 2004; Igamberdiev et al. 2004; Pandey et al. 2017) and an antiapoptotic function of HSPA9 and ACTA2 (Boldogh and Pon 2006; Gao et al. 2015). According to these results, sericin led to the recovery of cardiac mitochondrial function potentially by improving energy production via pyruvate metabolic activity and inducing cardioprotective activity via antioxidative stress and anti-apoptosis effects.

For the other three energy production pathways, including fatty acid oxidation (HSDL2, ACAT1, and ACADYL), the Krebs cycle (FH and ACO2), and the electron transport chain (SDHA, UQCRC2, NDUFA10, and ETFDH), the differentially expressed proteins were mixed in terms of upregulation and downregulation after sericin treatment in cardiac mitochondria under hypercholesterolemic conditions. Two proteins, ACAT1 and NDUFA10, were the most significantly upregulated proteins after sericin treatment. Immunofluorescence and immunogold labelling validation clearly revealed upregulated expression of ACAT1 and NDUFA10 in cardiac tissue and mitochondrial organelles, respectively. These data confirmed the correlation between protein expression and mitochondrial structure. In terms of function, ACAT1 is involved in the fatty acid oxidation pathway (Fukao et al. 1998). This enzyme plays a role in posttranslational modification-regulated mitochondrial protein activity (Still et al. 2013). In cardiovascular diseases, a lack of ACAT1 has been shown to increase the risk of atherosclerosis in a hypercholesterolaemia mouse model (Wakabayashi et al. 2018). Therefore, the increase in ACAT1 expression after sericin treatment may imply that the improved mitochondrial energy production is related to the fatty acid oxidation pathway and cardiovascular protection. Another protein, NDUFA10, functions in complex I of the electron transport chain and is involved in antioxidative stress. Silencing NDUFA10 results in a decreased number of mitochondria (Pandey et al. 2017). This finding supports our data, as upregulated NDUFA10 expression resulted in an increase in the number of normal-stage mitochondria. This result indicated that sericin upregulated NDUFA10 expression, leading to greater electron transport chain activity, increased antioxidative protection, and consequently recovered mitochondrial architecture.

In the Krebs cycle, downregulated ACO2 was observed after sericin treatment. Related reports have revealed that the activity of ACO2 relies on acetylation (Fernandes et al. 2015). It has been reported that decreased ACO2 levels in the heart are associated with cardiovascular diseases (Vivanco et al. 2003). In a plant study, aconitate hydratase (ACO) was reported to have antioxidative stress properties (Moeder et al. 2007). Hence, the effect of sericin, which reduced ACO2 expression, did not recover cardiac mitochondrial dysfunction under hypercholesterolemic conditions. In contrast, when observed in liver mitochondria, ACO2 has been shown to be upregulated by sericin and to maintain antioxidative stress activity (Ampawong et al. 2018). This result suggests that the Krebs cycle is not the specific pathway involved in the effects of sericin in cardiac mitochondria.

Regarding mitochondrial metabolism, the function of the amino acid metabolism pathway revealed that sericin led to upregulated GOT2 levels in cardiac mitochondria. GOT2 catalyses the transamination of L-aspartate and 2-oxoglutarate to oxaloacetate and glutamate in cardiac mitochondria (Hoffmann & Solter 2008). An increased level of GOT2 is associated with oxidative stress, leading to cardiovascular mortality (Zoppini et al. 2016). Sericin-induced GOT2 expression may be related to heart dysfunction. In contrast, in liver mitochondria, reduced GOT2 has been observed after sericin treatment, which is related to decreased liver injury from hypercholesterolaemia (Ampawong et al. 2018). Therefore, amino acid metabolism may reflect different activities depending on the organ affected by hypercholesterolaemia. In contrast, this study revealed reduced PCCB, which functions in mitochondrial metabolism and is a subunit of propionyl-CoA carboxylase (PCC). PCC is an active enzyme in the mitochondrial matrix that catalyses the conversion of propionyl-CoA to d-methylmalonyl-CoA (Jiang et al. 2005). Dysfunction of PCC is related to metabolic disorders that cause morbidity and mortality (Wongkittichote et al. 2017). A pathogenic PCCB gene (mutation) is related to cardiomyopathy in clinical reports (Bernheim et al. 2017). The relationship between the PCCB expression level and cardiovascular defects remains unclear. Therefore, reduced PCCB after sericin treatment may result from mitochondrial compensation by the cell. From the information regarding mitochondrial metabolism proteins in relation to mitochondrial function, it cannot be concluded that this group of proteins is related to the improved effects of sericin on the mitochondrial structure.

The effect of sericin on the mitochondrial structure was observed by performing a quantitative proteomic study of the expression of ACTA2. Additionally, the mitochondrial structure count showed an increasing number of normal-stage mitochondria in sericin-treated cardiac mitochondria compared with nontreated mitochondria. This information indicated that the effect of sericin improved mitochondrial structure by inducing ACTA2 expression. ACTA2 is transcribed by the nuclear isoform of mitochondrial RNA polymerase and is localised in the cytosol (Lee et al. 2011). A related study reported that actin-mitochondrial interactions are necessary for mitochondrial dynamics, movement, and distribution in the cell (Boldogh and Pon 2006). Deletion of ACTA2 has an effect on apoptosis (Cheng et al. 2018). From these data, upregulation of ACTA2 is related to improved mitochondrial structure and is implicated in the recovery of energy production and protection from apoptosis.

Some limitations of this study should be noted. First, the animal experiment only included female rats. This study was designed according to a related study by Ampawong et al. (2018). To avoid biological variation, no male rats were included in this study. Second, extracted mitochondrial proteins were pooled mitochondria isolated from the hearts of each treatment group. The proteomics analysis results that were used to assemble the mitochondrial protein profile combined mitochondrial structures from all stages. To validate the expression level of the mitochondrial structure, quantitative techniques, including immunofluorescence microscopy and immunogold coupled with transmitted electron microscopy, were used to calculate protein expression specific to mitochondrial structure.

## Conclusions

Sericin exhibited an effect on recovering cardiac muscle by restoring normal cardiac mitochondria from dysmorphic mitochondria under hypercholesterolaemia conditions. The adaptive mechanism of sericin in mitochondrial dynamics induced fusion protein expression, which consequently reduced apoptosis. The proteomics approach revealed several differentially expressed cardiac mitochondrial proteins affected by sericin treatment. Significantly changed proteins were correlated with changes in energy production, mitochondrial metabolism, and mitochondrial structure. However, the improved structure of mitochondria from sericin treatment revealed the influence of the differential proteins on fatty acid oxidation, the electron transport chain, pyruvate metabolism and mitochondrial structural proteins. These altered proteins led to therapeutic effects and cardiac mitochondrial protection under hypercholesterolemic conditions. The findings of this study provide information for future alternative treatments of cardiovascular diseases.
